# Growth Hormone (GH) and Gonadotropin-Releasing Hormone (GnRH) in the Central Nervous System: A Potential Neurological Combinatory Therapy?

**DOI:** 10.3390/ijms19020375

**Published:** 2018-01-26

**Authors:** Carlos G. Martínez-Moreno, Denisse Calderón-Vallejo, Steve Harvey, Carlos Arámburo, José Luis Quintanar

**Affiliations:** 1 Departamento de Neurobiología Celular y Molecular, Instituto de Neurobiología, Campus Juriquilla, Universidad Nacional Autónoma de México, Boulevard Juriquilla 3001, Querétaro 76230, Mexico; aramburo@unam.mx; 2 Departamento de Fisiología y Farmacología, Centro de Ciencias Básicas, Universidad Autónoma de Aguascalientes, Av. Universidad 940, Ciudad Universitaria, Aguascalientes 20131, Mexico; dcalderon@correo.uaa.mx; 3 Department of Physiology, University of Alberta, Edmonton, AB T6G 2H7, Canada; steve.harvey@ualberta.ca

**Keywords:** GH, GnRH, neuroprotection, neuroregeneration, neurotrophic, therapy, CNS, extrapituitary, extrahypothalamic

## Abstract

This brief review of the neurological effects of growth hormone (GH) and gonadotropin-releasing hormone (GnRH) in the brain, particularly in the cerebral cortex, hypothalamus, hippocampus, cerebellum, spinal cord, neural retina, and brain tumors, summarizes recent information about their therapeutic potential as treatments for different neuropathologies and neurodegenerative processes. The effect of GH and GnRH (by independent administration) has been associated with beneficial impacts in patients with brain trauma and spinal cord injuries. Both GH and GnRH have demonstrated potent neurotrophic, neuroprotective, and neuroregenerative action. Positive behavioral and cognitive effects are also associated with GH and GnRH administration. Increasing evidence suggests the possibility of a multifactorial therapy that includes both GH and GnRH.

## 1. Introduction

It has been understood that growth hormone (GH) and gonadotropin-releasing hormone (GnRH) mainly affect the pituitary gland; however, in the last 20 years several studies have started to change this notion. Now, it has become clear that several hypothalamic and pituitary hormones, including GnRH and GH and their corresponding canonic receptors, have ubiquitous expression and a diversity of biological functions. There is increasing evidence indicating that these hormones are expressed in extrapituitary and extra-hypothalamic tissues and are involved in local action, which may include effects on reproduction, growth, cell survival, tissue repair and renewal, immunomodulation, metabolism, neural function, and neuroregeneration [1,2,3,4,5,6].

It is known that GH is mainly produced by the pituitary somatotropes and is secreted as an endocrine hormone that regulates growth and differentiation during development [7]. Postnatally, GH pulsatile release is required as a homeostatic factor that in many tissues is critical for the maintenance of their metabolic actions, as well as cell proliferation and differentiation. GH may exert its effects either directly by the activation of signaling pathways after binding to the GH receptor (GHR), or indirectly through its classical mediator the insulin-like growth factor-I (IGF-I) [8]. In recent years, it has been reported that GH is also produced in several tissues and cell types, in addition to the pituitary somatotropes. Thus, it is now accepted that GH may act not only as an endocrine hormone, but is capable of exerting paracrine, autocrine, and even intracrine actions in extrapituitary locations such as tissues belonging to the reproductive, immune, and nervous systems [5]. However, the mechanisms that regulate the fine equilibrium between endocrine and autocrine/paracrine GH and their relative participation and implications in GH physiology still remain largely unknown.

It is widely known that specific hypothalamic neurons synthesize and release GnRH, a bioactive decapeptide that is secreted by nerve terminals at the median eminence and travels through the hypophyseal portal blood vessels to bind the GnRH receptor (GnRH-R) in pituitary gonadotropes. GnRH stimulation of gonadotropes is required for the biosynthesis of luteinizing hormone (LH) and follicle-stimulating hormone (FSH), which have specific biological actions upon the gonads to regulate gametogenesis and steroidogenesis [9]. Recently, the presence of GnRH-R, as well as the expression and secretion of GnRH in extrapituitary tissues, have been described, although little information exists regarding the function of these receptors and the hormone itself in these locations [1]. 

This review is focused on the effects of GH and GnRH (or agonists) in the nervous system and their possible application as neurotrophic factors in different neurological pathologies. Currently, there are clinical trials underway to research the neurotrophic actions of both GH and GnRH in patients with brain trauma and neurodegenerative diseases [10]. It is important to mention that children and adults treated with GH as a replacement therapy for GH deficiency (GHD) have shown cognitive and motor function improvement [11]. However, to date, the simultaneous application of GH and GnRH has only been studied in patients with short stature and infertility [12,13,14], but its potential as a combinatory neurotrophic therapy for neural damage or neurodegenerative diseases remains completely unknown.

## 2. Expression and Neurotrophic Effects of Growth Hormone (GH) in the Central Nervous System (CNS)

Growth hormone (GH) is a key factor during nervous system development, acting as a strong promoter of growth and differentiation; these actions may be exerted either directly by activation of the GH receptor (GHR) or indirectly through its classical mediator, IGF-I [15]. It is now well established that GH is synthesized in many extrapituitary tissues that originally were described as the target of its actions [5]. Neural tissues are now known as a site of GH expression, where the local hormone production coexists with the GH arriving through the circulation, indicating overlapping effects of GH, mediated by autocrine/paracrine and endocrine mechanisms, occurring just after the onset of the somatotrophs during embryonic development [7]. Both locally produced and systemic GH, are necessary to provide complete and on-time development; this coexistence also suggests the important actions of GH in neural function during adult stages. During development, GH plays a pivotal role in proper brain growth; GHD children have reduced volume in structures such as the corpus callosum, hippocampus, thalamus, and basal ganglia, which correlates with cognitive and motor function deficits [16]. The effect of GHR dysfunctions (GHRD) in cognition is a controversial topic; however, the incidence of mental retardation in children with Laron syndrome is significantly higher [17,18,19,20]. Cognitive function deficits associated with GH deficiency (by aging, traumatic brain injury, or endocrine disorder) can be improved with GH replacement [11,21,22,23,24,25,26,27]. There are conclusive data about the positive effects of GH on cognitive function and behavior [10,28]. The widespread presence of GHR in the CNS and the neurotrophic effects of GH, together with many reports of its novel clinical application to treat neurodegenerative diseases including Alzheimer’s disease, amyotrophic lateral sclerosis, Parkinson’s disease, and brain trauma (reviewed in Bianchi et al. [10]), clearly imply a therapeutic potential that is worthy of research and review (Table 1).

### 2.1. Cerebral Cortex

GH immunoreactivity in the cerebral cortex has been described in primate, rodent, and chicken brains [6,75,76,77]. The fact that GH crosses the blood–brain barrier (BBB) under normal and physiopathological conditions [28,78,79] suggests that both endocrine and autocrine/paracrine mechanisms could be involved in its action in the cerebral cortex. 

In the rat and rabbit, the presence of GHR was found in several layers of the cerebral cortex by immunohistochemistry and in situ hybridization [80]. It was found that GH immunoreactivity increased in cortical neurons after hypoxic–ischemic brain injury, and also that treatment with GH right after he insult diminished the extent of neuronal loss [77]. In GHR null mice (GHR−/−), adult brain size is diminished; however, the density of cortical interneurons positive for calreticulin and calbindin increased [81]. Moreover, a decrease in the cortical neuropil and the neurite trees was abnormal in GHR mutants [81]. It has been suggested that this neuroprotective effect of GH might be exerted directly by the GHR without the mediation of IGF-I [77]. In the brain cortex, GH bioactivity is mediated by the Janus kinase/Signal Transducer and Activator of Transcription JAK/STAT pathway, as observed in treated rats in which pSTAT immunoreactivity increased in neurons but not in glial cells [82]. The specific binding of GH to the GHR present in the cerebral cortex was demonstrated by incubation of ^125^I-labeled rat GH with cerebral cortex homogenates [83]. GHR activation induces an increase in cell proliferation in cortical progenitor cells obtained from rat embryos [29]. Using an LUC system with the IGF-I promoter, a luminescence increase was observed in the cerebral cortex of mice injected with GH, which demonstrates, as in many other tissues, the involvement of locally IGF-I mediated indirect effects [84]. In recent years, Walser et al. [85] showed that bGH induced changes in GH-related genes (*Ghr*, *Igf1*, *IgfIr*, and *Esr1*), neuron-related genes (*Psd95*, *Gabab1*, *Gria1*, *Nr2a*, and *Hbb*), and glia-related genes (*Cx43*, *Gfap*) in the male parietal cortex. In the female cerebral cortex, the mode of administration of GH (episodic versus continuous) showed a differential response of the *Hbb* and 5′-aminolevulinate synthase (*Alas2*) expression [86]. In the cerebral cortex, GH bioactivity was associated with cognitive function and behavior modulation through the activation of genes that showed dimorphic sexual response. 

Neurotransmission is modulated by GH actions, as GABA B receptors responded to its administration in the cingulate cortex, primary motor cortex and the caudate putamen, and this effect was correlated with an increase in cognitive function [47,48]. There were clear implications of GH in the opioid system function in which it was able to decrease levels of the delta-opioid receptor in layers I–IV of the cerebral cortex [87]. In brain trauma, GH has demonstrated neurotrophic actions in the cognitive, sensorial, and motor functions [57,58,88,89]. In an experimental model for brain contusion recovery, GH improved cognitive function and increased the brain-derived neurotrophic factor (BDNF) and TrkB in the prefrontal area [73]. GH effects in cortical areas are documented in normal neural function as well as after a neural injury. 

The effects of GH in the brain cortex are not restricted to the neural linage since it is able to activate GHRs present in the glial cells [80]. The reduction of cortical and subcortical brain areas observed in GHR−/− mice is associated with a decrease in the number of glial cells positive for glial fibrillary acidic protein (GFAP) [81]. In addition, GHR activation in the brain cortex increased connexin-43 (gap junction protein); interestingly, this action was restricted to some specific brain areas since it did not activate glial cells in the hippocampus and brainstem [90]. 

### 2.2. Subcortical Organs

#### 2.2.1. Hypothalamus

The expression of GH and GHR in the hypothalamus is involved in the control of energetic balance and metabolism [30,31]. It is now accepted that endocrine and hypothalamic GH interact to regulate metabolic function through the hypophysiotropic factors controlling the anterior pituitary gland. Hypothalamic GH-immunoreactive neurons projecting to the median eminence were retrograde-labeled; in addition, GH mRNA was also localized in these cells [91]. GH gene expression in the lateral hypothalamus significantly increased in response to GHRH and decreased under stress; this GH mRNA modulation suggests a physiological effect of this extrapituitary production [92]. In addition, hypothalamic GH expression has been shown to be responsive to estradiol and also shows sexual dimorphism [91,93].

In the rat hypothalamus, GH administration induces a strong phosphorylation of STAT5 in the supraoptic, paraventricular, suprachiasmatic, periventricular, arcuate, ventromedial, dorsomedial, tuberal, posterior, and ventral premammillary nuclei [82]. GHR expression in the arcuate nucleus is related to a negative feedback loop that regulates GHRH and somatostatin release to the pituitary gland [94,95]. Moreover, systemic GH administration increases c-fos and neuropeptide Y (NPY) in the rat hypothalamus [96]. In addition to the neurons in the NPY circuit that express GHR, a leptin receptor (LepRb) circuit also expressing GHR has recently been described, and it has been proposed that this nutrient sensor system controls hepatic glucose independently of feeding behavior [31]. Activation of GHR by GH overexpression in the mouse hypothalamus has a powerful orexigenic effect [74]. Furthermore, a transgenic model of GH overexpression in GHRH- and vasopressin neurons in the hypothalamus expressed a dwarf rat phenotype, showing that local production is able to decrease the positive effects of the releasing factors in the pituitary gland [97,98].

#### 2.2.2. Cerebellum

Cerebellar function is critical for the survival of all vertebrates. The coordination and regulation of muscular motor function is a well-developed and conserved system that plays an important role in evolution. The presence of GH receptors in this structure and the significant GH-specific binding that has been reported suggest a physiological function not only during development but also in adult cerebellar function [32,75,83,99]. Growth hormone deficiency is associated with Joubert syndrome, a ciliopathy defined by cerebellar abnormalities caused by a mutation in the KIAA0753 complex; this pathology shows a hypoplastic cerebellar vermis during fetal development that leads to malformations in the midbrain and cerebellum [33]. Thus, it has been implied that GH action is a necessary component of proper cerebellar development.

The cerebellum is not only a site of GH action, since there is evidence that both GH mRNA and protein are present in Purkinje cells, granular cells, and some cells in the molecular cell layer, which implies autocrine and paracrine physiological action [60,76]. GH expression in the rat cerebellum seems to be influenced by sex steroids, where estradiol is able to increase its mRNA levels both in males and females [93]. In the chicken cerebellum, GH has been shown to exert neuroprotective actions against a hypoxia–ischemia injury, both in vivo and in vitro [61]. These effects are due to GH anti-apoptotic mechanisms that include an increase of Bcl-2 expression and the activation of the PI3K/Akt pathway, as demonstrated in cerebellar primary cell cultures [61]. The influence of GH through pro-survival and anti-apoptotic actions is likely to be involved in the high resistance of this brain structure to damage. In a similar manner to cortical areas, GH decreased opioid receptors in whole brain extracts and cerebellum [87,100]. It is very likely that the improvement in the fine-tuning control of movement observed in Down’s syndrome patients treated with GH involves the action of GH and IGF-I in the cerebellum [101,102,103].

#### 2.2.3. Hippocampus

The hippocampus is well known as a site of action of GH; its main effects include improving cognitive function, memory, and learning [76]. Hippocampal neurons are also known for having high renewal and this neural renovation, particularly in the subgranular zone (SGZ), has been associated with GH and local IGF-I actions; however, neurogenesis is even more active in the subventricular zone (SVZ) [38,39,40,41,104,105]. The strong action of GH in the renewal, differentiation, and migration of neural stem and progenitor cells in the SVZ has been reported in murine models, both in vivo and in vitro [41,42,43,44,45,46]. The anti-apoptotic actions of GH are well established in the hippocampus and these protective effects are effective against drug and alcohol damage [62,63,64]. The neuroprotective actions of GH in the hippocampus include prevention of excitotoxic cell death [106]. In lactating rats treated with kainic acid, hippocampal GH increased significantly, demonstrating a locally mediated neuroprotection mechanism [107]. It has been shown that damage due to oxidative stress, neurotoxicity, or hypoxic injury was reduced in GH-treated subjects, suggesting that this effect likely involves both local and systemic IGF-I actions [108,109,110].

Hippocampal neurotransmission is improved by GH administration, facilitating the excitatory activity of glutamate receptors (AMPA and NDMA) in both young and old rats and enhancing basal and long-term potentiation (LTP) [49,50,51,52,111]. LTP was improved in rats treated with GH by intrahippocampal administration after a nucleus basalis injury, possibly through hormonally induced neurogenesis [112]. The clinical application of GH in patients with memory and cognitive dysfunction is currently being researched; however, there are some clinical and experimental data showing the potential of its application [26,113,114,115]. Conversely, a recent report from Basu et al. [116] showed that transgenic (Tg) mice overexpressing bovine GH have more latency and errors in a spatial learning test in comparison to the control group; in contrast, Tg mice expressing a GH-receptor antagonist showed better performance than wild-type animals.

Brain damage provoked during delivery was treated with GH in a 10-year-old girl, resulting in a significant improvement in cognitive function [117]. In patients treated with opioids, GH improved hippocampal cognitive function [118]. This beneficial effect likely involves the downregulation of NMDA receptors and the prevention of opioid-related excitotoxicity [64]. It has been proposed that GH can modulate NMDA and AMPA receptors through the mitogen-activated protein kinases/extracellular singal-related kinases (MEK/ERK) pathway, exerting positive effects in postsynaptic neurons in the *Cornus Ammonis* 1 (CA1) [119].

Similar to what happens in other brain areas, the hippocampus is a site of extrapituitary GH expression [120,121], which also leads to the notion that autocrine and/or paracrine regulatory mechanisms are involved in these actions. Interestingly, it was shown that GH expression in the amygdala, a functional related structure contiguous to the hippocampus, increased under chronic fear and during fear learning conditions [122]. Also, in the male rat hippocampus, a strong response in glial-related and neural globin (*Hbb*) gene expression was observed after GH treatment under both episodic and continuous administration schemes [84]. On the other hand, neural- and glial-related genes also responded to GH in the female hippocampus, demonstrating an activation of both neural and glial cells [86].

### 2.3. Spinal Cord

GH and GHR immunoreactivities in the spinal cord are present during embryonic development and also in adult rats and chickens [7,95,123,124]. Administered GH was able to bind specifically to the GHR present in the rat spinal cord [125]. Rats treated with a GH antagonist (G129R) resulted in poor development of the spinal cord, which indicates a physiological implication during the central nervous system (CNS) growth [126]. Postnatal growth hormone deficiency in rats caused a decline in the activity of spinal cord acetylcholinesterase, but not butyrylcholinesterase, which implies that GH does not affect the development of non-neuronal elements as much as it does the neuronal and synaptic compartments of the developing rat spinal cord [127].

In patients with spinal cord trauma, systemic GH and IGF-I levels were significantly reduced and showed a lower response to GHRH [128,129,130]. In contrast, a significant increase in plasmatic GH was observed one week after spinal cord injury [131]. In addition, a spinal cord injury increases the vascular permeability of GH as a possible emergency mechanism [132]. Lumbar moto-neurons are responsive to GH, in which GH overexpression increased nucleolar, nuclear, and cell body size [53]. Hanci et al. [133] reported that GH treatment attenuated the motor dysfunction derived from spinal trauma in rats. Macroscopic and microscopic negative effects of radiation on the spinal cord were prevented in rats treated with systemic injections of GH [134]. Moreover, topical application of GH within a short post-injury time frame showed an induction of neuroprotection after spinal cord injury (SCI) [65,66], and it is thought that this protective GH action is likely to also involve the expression of neurotrophins such as BDNF, GDNF, and IGF-I [67,68]. A nanowired delivery of GH that extends the short hormone lifetime was applied intrathecally and resulted in increased neuroprotection after an SCI [69]. Additionally, GH was demonstrated to alleviate pain through an increase of IGF-I in the root dorsal ganglia [135]. A recent report of a clinical case with caudal regression syndrome, which is characterized by incomplete spinal cord development, was treated with hGH and resulted in significant improvement in motor function, suggesting an innervation of distal sections of limbs [136]. GH actions promoting innervation, neurogenesis and neuroregeneration are widely accepted [34,59], however the molecular and cellular mechanisms associated to these actions in the brain and spinal cord are still largely unexplored.

## 3. Expression and Neurotrophic Effects of GnRH in the CNS

There is increasing evidence about the actions of GnRH in neural tissue, including neurotrophic, neuroprotective, and neurodegenerative (Table 2). These recently described GnRH neural actions could lead to novel therapeutic application in order to prevent and treat different neurodegenerative processes underlying pathologies and trauma of the nervous system.

### 3.1. Cerebral Cortex

There is a widespread incidence of GnRH and GnRH-R immunoreactive neurons in the cerebral cortex, suggesting that GnRH may act as a common neuromodulatory peptide [145]. The presence of a GnRH receptor and expression of its mRNA was demonstrated by immunohistochemistry and RT-PCR analysis in both cerebral cortical neurons of rat embryos and cerebral cortical tissues of adult rats. In addition, a decrease in GnRH-R mRNA expression was shown when cultured neurons of rat embryos were treated with GnRH [137].

In experiments carried out in rat cultured cortical neurons, it was observed that incubation with GnRH induced an increase in the expression of two cytoskeletal proteins, 68 and 200 kDa neurofilaments (NFs), as well as in the number and length of neurites [2]. Also, it was reported that the human cerebral cortex may contain low levels of GnRH [164] and that exogenous GnRH can access the brain [165]. There is evidence, in women, that chronic administration of leuprolide acetate (LA), a GnRH agonist, alters the neural circuitry underlying performance of visual working memory [159,160], but these studies cannot discriminate between direct LA effects or the induced hypo-oestrogenic environment.

On the other hand, in a recent study, Maeng et al. [161] found that the acute administration of LA enhanced extinction memory and was correlated with an increase in serum testosterone levels and increased c-fos activity within the infralimbic cortex in rats. These results suggest that the elevation in testosterone induced by acute administration of LA can influence extinction memory consolidation, perhaps through modification of neuronal activity within the infralimbic cortex. However, the mechanism of action is unclear, although there is evidence that incubation of rat brain cortex neurons with GnRH alone (without estrogens) has direct neurotrophic effects, stimulating neural outgrowth and NFs expression [2].

### 3.2. Subcortical Organs

#### 3.2.1. Hypothalamus

Using an electrophysiological approach, it was observed that the pattern of excitation in neurons of slices from the preoptic area was modified when they were perfused with LHRH [146,147]. This result is in keeping with the findings on the presence of GnRH-R in neurons in the preoptic area and arcuate nucleus [166,167]. In the case of the presence of GnRH-R in the GnRHergic neurons under physiological conditions, it has been proposed that GnRH may modulate its own release through an ultra-short loop feedback system [138]. Autocrine mechanisms of GnRH neurons possibly are involved in generating and modulating pulsatile release; these local actions could also be involved in the switch between pulse and surge modes of secretion [139,147,148].

#### 3.2.2. Cerebellum

Earlier studies demonstrated for the first time the presence of GnRH in the cerebellum [168,169]. Recently, the presence of GnRH-R immunoreactivity has been reported in the cerebellum, specifically in the middle lobe of the human cerebellum [3] and in the Purkinje cells of mice cerebellum [166,170]. Cerebellar Purkinje cells are GABAergic and provide inhibitory output from the cerebellum, while cerebellar granule cells act to regulate the actions of the Purkinje cells through excitatory glutamatergic input [149]. GnRH administration significantly affects both cerebellar glutamate and GABA content [150]. It is thus plausible that GnRH may act as a neuropeptide on the Purkinje cells to regulate their interactions [166].

An important issue is to determine the way in which GnRH can reach the cerebellum and produce its neuroendocrine effects. Although unlikely, considering the distance of the cerebellum from the ventricles, it is possible that some GnRH may access these cerebellar neurons through the cerebrospinal fluid [145].

#### 3.2.3. Hippocampus

Several studies indicated that high densities of GnRH-R were present in CA1–CA3 regions of the hippocampus [151,162,171]. Moreover, it has been shown that GnRH-R activation in the hippocampus could be associated with sexual behavior regulation in mammals [162]. Jennes [172] found that intracerebroventricular injections of GnRH caused an increase in the number of c-fos-positive cells in CA1 and CA3 areas. The induction of c-fos synthesis after GnRH administration was transient and reached a maximum after 1 to 2 h, before it declined to pretreatment levels after 8 h. These results suggest that GnRH exerts specific effects on protein synthesis in certain neurons of the hippocampus and that these effects are, at least in part, mediated by c-fos. Lu et al. [162] reported that activation of GnRH-R produced a long-term increase in intrinsic neuronal excitability in both the CA1 and CA3 pyramidal neurons of rat hippocampus. Furthermore, Yang et al. [152] found that GnRH-R activation induced sustained enhancement of synaptic transmission, mediated by ionotropic glutamate receptors in CA1 pyramidal neurons. This synaptic potentiation was associated with the stimulation of protein kinase C (PKC). Likewise, it has been described that GnRH stimulates sexual neurosteroids (NS) synthesis in the hippocampus. The upregulation of NS synthesis was paralleled by a dose-dependent increase in synapse number in in vitro studies [151]. An increase in synapse number was also seen in vivo after the infusion of GnRH into the hippocampus [173]. A higher synapse density has also been observed, as well as an increase in spinophilin immunoreactivity, in hippocampal cultures incubated with GnRH [153]. Thus, it is possible that stimulation of the hippocampus with GnRH can modify functions such as reproductive behavior, emotions, and memory, among others [163].

### 3.3. Spinal Cord

It has been shown that the adult sheep spinal cord expresses GnRH and GnRH-R as well as their corresponding mRNAs [174]. In another study, the presence of GnRH-R and its mRNA expression was found in spinal cord neurons of rat embryos and adult rats [140]. Additional in vitro experiments showed that the expression of GnRH receptor mRNA decreased in spinal cord neurons exposed to GnRH in comparison to unexposed ones, indicating a downregulation of the receptor [140]. This finding suggests that this receptor is sensitive to its natural agonist (GnRH) and there is a spinal mechanism involved in the regulation of GnRH receptor expression, similar to that occurring in pituitary gonadotropes [175].

**Figure 1:**
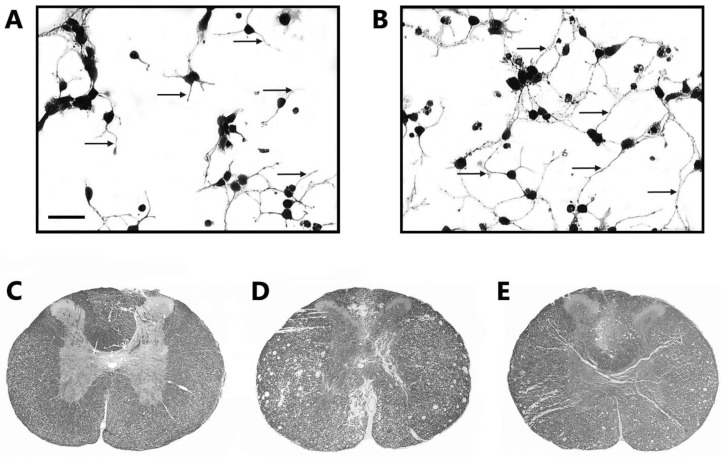
Neurotrophic effects of gonadotropin-releasing hormone (GnRH) or its agonist, leuprolide acetate, on the rat spinal cord. (**A**) Spinal cord neurons in culture incubated with saline; (**B**) treated with GnRH (10 nM) for 24 h; (**C**) spinal cord section of control rats; (**D**) injured spinal cord of rats that received saline solution; (**E**) spinal cord of rats treated with leuprolide acetate (10 µg/kg, i.m.) for five weeks. In spinal cord sections of rats treated with leuprolide acetate, the configuration of white and gray matter is more similar to that in the control rats and looks better than in those that received saline solution only. Spinal cord injury sections from rats with saline solution show many large cavities without nervous tissue. 10× magnification. The arrows indicate neuritic growth. Bar scale is 10 µM.

In rat embryos, Quintanar et al. [154] studied the effects of GnRH on neurites and upon 68 and 200 kDa neurofilament expression in spinal cord neurons in culture; in addition, changes in spinohilin gene expression were also determined (spinophilin is considered a marker of synaptic contact). The results showed that GnRH stimulates neurite outgrowth (Figure 1A,B) in addition to an increase in neurofilaments and spinophilin expression. Furthermore, in experiments performed on castrated animals with spinal cord injury it was observed that the administration of GnRH increased the number and caliber of nerve axons of the spinal cord, and in the case of white matter, spared tissue was significantly higher than in the control animals treated with saline solution. Likewise, the expression of spinophilin in the spinal cord was slightly increased with respect to those not treated. Associated with these neurological changes, it was also found that those animals treated with GnRH significantly increased the length and velocity of their stride compared to the non-lesioned control group [157].

In another study, rats with spinal cord compression injury were treated with the GnRH agonist LA. They showed locomotor activity recovery, improvement in kinematic gait, and voiding reflex recovery of the bladder. Furthermore, LA treatment induced a greater conserved area of white and gray matter in the spinal cord, similar to that observed with GnRH treatment (Figure 1C–E) [158]. Similar results have been obtained in rats with experimental autoimmune encephalomyelitis, where LA decreased clinical signs of disease and increased expression of neurofilaments and axonal caliber in the spinal cord [155]. These findings suggest that GnRH, or its analogue, LA, may play a role as a neuromodulator in neuronal plasticity and could be considered a potential factor for neural regeneration in spinal cord injuries.

## 4. GH and GnRH Effects on Other Neural Tissues

### 4.1. Neuroretina

The retina is a site of expression of many hormones, releasing factors, growth factors, and neuropeptides. Among this high diversity of autocrine and paracrine messengers, GH and GnRH, together with their respective receptors, are expressed in the retina [54,141,142]. This non-canonical distribution and expression has been described mainly in non-mammalian species, and there is increasing interest in their neurotrophic actions as an alternative when treating neurodegenerative diseases in the retina.

**Figure 2:**
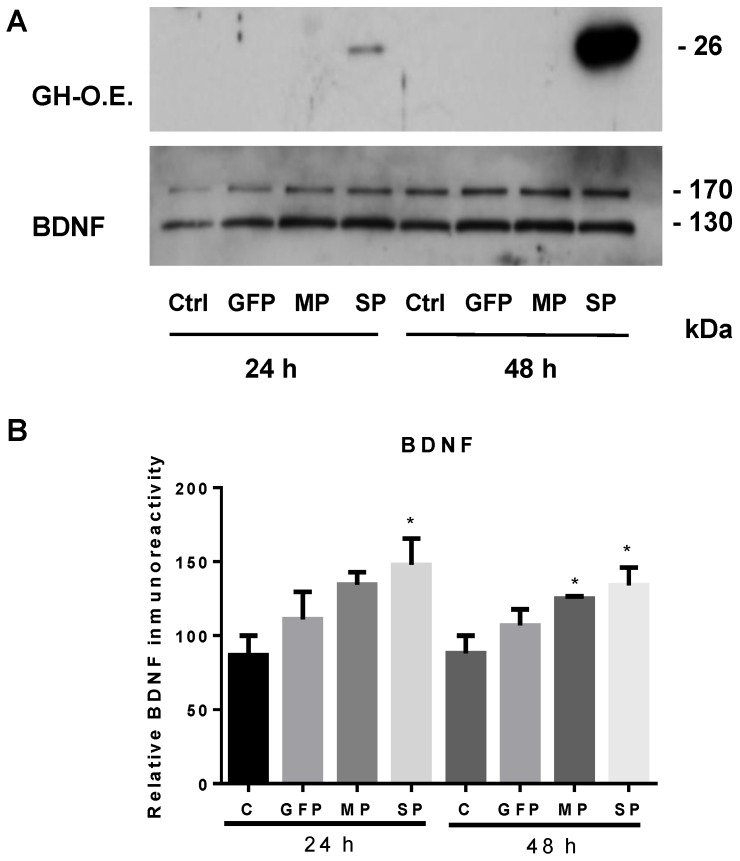
Effects of growth hormone (GH) over-expression on brain-derived neurotrophic factor (BDNF) secretion in neuroretina-derived quail cell line (QNR/D) cell cultures. Culture media (20 µL) were analyzed at 24 and 48 h post-transfection by Western blot (reducing conditions). (**A**) Representative luminograms of GH (**top**) and BDNF (**bottom**) in the culture media; (**B**) relative changes in BDNF immunoreactivity were determined by densitometry (*n* = 3). Control (C; not transfected) and green fluorescent protein (GFP; pCAG plasmid by Addgene) overexpression groups were used as controls. MP (mature peptide) is a plasmid construction expressing a non-secreted GH and the SP (signal peptide) construction that produces a secreted GH. Transfections were performed as reported in [71]. Asterisks show significant differences (*p* < 0.05) in comparison to the control (C), as determined by one-way ANOVA and Tukey post hoc test.

GH is an important factor during retinal growth and regulates equilibrium between developmental apoptosis and proliferation waves [35,36]. The anti-apoptotic and pro-survival actions of GH were tested against the overactivation of glutamate receptors, providing interesting evidence about its neuroprotective actions in lower vertebrates [70,71]. GH actions in the retina are mediated by its receptor GHR and involve the activation of the JAK/STAT and PI3K Akt pathway, and a Bcl2 increase [71]. The anti-apoptotic actions in the retina involve Trks and ERK pathways, which converge in the activation of cAMP and resulted in a decrease of caspase-3 and -9 activities [176]. Moreover, systemic injections of GH can cross the retinal blood barrier (RBB) and reach the retinal ganglion cells, where it is internalized by the cells, resulting in neurotrophic action [55]. The presence and action of GH upon the survival of RGCs was also observed in rats and humans, where it seems to work as a homeostatic factor under physiological conditions [177,178]. The relation of abnormal levels of GH to the etiology of eye developmental diseases, retinal neurodegenerative processes, and vision deficiency is still poorly understood; however, there is conclusive information about the somatotropic influence in several retinal physiopathologies that demonstrate its participation as a key regulator of cell survival, homeostasis, and metabolism [179,180]. Exogenous GH is able to induce an increase of NT3 and BDNF expression in neuroretinal cells, which correlates with neurite growth in control and excitotoxic conditions [72]. Interestingly, retinal GH induces autocrine/paracrine and intracrine effects in these cells, since the overexpression of a non-secreted GH variant is also able to induce BDNF release (Figure 2) and neuroprotection [71]. These intracrine effects open up the possibility of expressing GH in specific cell types without generating side effects in non-target cells.

GnRH-R gene promoter activity was detected in the rat retina during embryonic development, implicating its action in the eye growth and differentiation [141]. In the zebrafish, GnRH signaling is required for proper axon projection of terminal neurons, and these cells synapse with interplexiform cells and retinal ganglion cells (RGCs) [156]. Recently, a report from Corchuelo et al. [142] demonstrated that GnRH is involved in the neuromodulation of the retina, particularly in the final stages of maturation. Retinal GnRH in the European sea bass is proposed to act as a modulator of dopaminergic cell activity [181]. Witkin reported [182] the presence of LHRH immunoreactivity in the optic nerve of the fetal rhesus macaque and in other primates. A similar LHRH immunoreactivity distribution was reported in the optic nerve, optic chiasm, and optic tract of the adult rat [183]: the presence of LHRH in the axons of RGCs shows a potential modulating effect in mammal vision [184].

### 4.2. Brain Tumors

The presence of GH and GnRH receptors has been reported in tumor cell lines of neural origin and brain tumors [37,144]. However, it should be kept in mind that the expression of hormonal receptors is not necessarily consistent, even in the same type of tumors [37,143,185]. Both GH and GnRH could be potential inducers of tumor growth [186,187], which must be a major consideration for the application of these two peptide factors as a treatment for any neural disease. In addition, there is evidence that brain cancer cells are able to produce GH or GnRH [37,143]. This leads to the hypothesis that they can respond to the endocrine/paracrine influence of this peptide, and also that the tumor has the capacity to produce its own hormones.

Growth hormone receptors are present in many cancer tumors including brain tumors; the expression of GHR has been reported with particularly high incidence in glioblastoma cell lines and gliomas [186]. There is contradictory evidence about the carcinogenesis, progression, and recurrence processes of brain tumors in patients treated with GH [188,189]. However, in vitro, evidence shows that GH is able to induce IGF-I, and this growth factor is able to promote the proliferation and migration of brain cancer cells. In addition, GH is able to induce axonal growth in neuroblastoma cells [56]. Moreover, GH, GHR, and IGF-I have been detected within the same glioma cell, suggesting autocrine/paracrine actions that could be related to the resistance and aggressive behavior of this type of cancer [37]. To date, there is a lack of information about the contribution, regulation, and molecular mechanisms of local growth factors and hormones involved in brain tumors.

Autocrine and paracrine actions of GnRH through the GnRH-R in nervous system tumors have been observed in meningiomas and low-grade glioblastoma multiforme, and it has been proposed that GnRH-R could be used as a prognosis marker [143,144]. Interestingly, the expression of GnRH-R in many cancer cell types leads to the strategy of using it as a way to induce specific cytotoxicity through AN-152 (AEZS-108), a toxic analog of GnRH, which is internalized after binding to the receptor and subsequently accumulates until it induces cell death [190]. It is clear that hormones such as GH and GnRH have a relationship with the pathophysiology of nervous system cancer that needs to be investigated.

## 5. Conclusions

The therapeutic application or blockade of growth factors, neuropeptides, hormones, and cytokines is increasing as a treatment for multiple neural injuries, deficits, and dysfunctions. Despite the fact that GnRH and GH now have accepted roles in neuromodulation, neuroprotection, and neuroregeneration in different areas of the nervous system, there is a lack of information about the molecular and cellular processes underlying these actions, which include gene multiplicity, receptor cross-talk, signaling pathways, and target genes. The immune system and vascular system within the nervous system, in which GnRH and GH ligands and receptors are also expressed, are very important players in this game that have not been included in this review but whose interactions are of crucial relevance. The potential combinatory effect of GH and GnRH in the nervous system, particularly after neural damage, has not been thoroughly investigated, but based on the evidence reviewed here it is likely that additive and synergistic effects could be observed.

## Figures and Tables

**Table 1:** Neural effects of growth hormone (GH) in the nervous system.

Structure/Effect	Cortex	Hypothalamus	Cerebellum	Hippocampus	Spinal Cord	Neuro Retina	Brain Tumors
Structure Development	+ [29]	+ [30,31]	+ [32,33]	+ [34]	+ [7]	+ [35,36]	+ [37]
Proliferation & Differentiation	+ [29]	n/d	+ [32]	+ [38,39,40,41,42,43,44,45,46]	n/d	n/d	n/d
Axon/Dendrite growth & Synaptic actions	+ [47,48]	n/d	+ [32]	+ [49,50,51,52]	+ [53]	+ [54,55]	+ [56]
Neuroprotection & Neuroregeneration	+ [57,58,59]	n/d	+ [32,60,61]	+ [62,63,64]	+ [65,66,67,68,69]	+ [70,71,72]	n/d
Cognitive & Behavior	+ [73]	+ [31,74]	+ [33]	+ [57]	n/d	n/d	n/d

n/d: not determined; +: actions of GH were reported.

**Table 2:** Neural effects of GnRH in the nervous system.

Structure/Effect	Cortex	Hypothalamus	Cerebellum	Hippocampus	Spinal Cord	Neuro Retina	Brain Tumors
Structure Development	+ [137]	+ [138,139]	n/d	+ [138]	+ [140]	+ [141,142]	+ [143,144]
Proliferation & Differentiation	n/d	n/d	n/d	n/d	n/d	n/d	n/d
Axon/Dendrite growth & Synaptic actions	+ [2,145]	+ [139,146,147,148]	+ [149,150]	+ [151,152,153]	+ [154,155]	+ [142,156]	n/d
Neuroprotection & Neuroregeneration	+ [2]	n/d	n/d	n/d	+ [155,157,158]	n/d	n/d
Cognitive & Behavior	+ [159,160,161]	n/d	n/d	+ [162,163]	n/d	n/d	n/d

n/d: not determined; +: actions of GH were reported.
